# The Stability of Metal Halide Perovskite Nanocrystals—A Key Issue for the Application on Quantum-Dot-Based Micro Light-Emitting Diodes Display

**DOI:** 10.3390/nano10071375

**Published:** 2020-07-15

**Authors:** Zhibin Shangguan, Xi Zheng, Jing Zhang, Wansheng Lin, Weijie Guo, Cheng Li, Tingzhu Wu, Yue Lin, Zhong Chen

**Affiliations:** School of Electronic Science and Engineering, Xiamen University, Xiamen 361005, Fujian, China; shangguanzhibin@stu.xmu.edu.cn (Z.S.); zhengxijjyz@163.com (X.Z.); jingzhang@stu.xmu.edu.cn (J.Z.); wslin@stu.xmu.edu.cn (W.L.); wjguo@stu.xmu.edu.cn (W.G.); chenz@xmu.edu.cn (Z.C.)

**Keywords:** micro-LED, perovskite nano crystal, stability

## Abstract

The metal halide perovskite nanocrystal (MHP-NC), an easy-to-fabricate and low cost fluorescent material, is recognized to be among the promising candidates of the color conversion material in the micro light-emitting diode (micro-LED) display, providing that the stability can be further enhanced. It is found that the water steam, oxygen, thermal radiation and light irradiation—four typical external factors in the ambient environment related to micro-LED display—can gradually alter and destroy the crystal lattice. Despite the similar phenomena of photoluminescence quenching, the respective encroaching processes related to these four factors are found to be different from one another. The encroaching mechanisms are collected and introduced in separate categories with respect to each external factor. Thereafter, a combined effect of these four factors in an environment mimicking real working conditions of micro-LED display are also introduced. Finally, recent progress on the full-color application of MHP-NC is also reviewed in brief.

## 1. Introduction

The micro-LED display would probably become the next generation display technology if those major challenges it currently faces were conquered [1,2]. The most pivotal one among them is the full-color solution, i.e., the way the display shows different colors. There have been two mainsteam full-color solutions: (a) The tricolor-chips solution, in which three prime colors (RGB) of an individual pixel are directly generated from a set of red, green and blue micro-chips; (b) The color converter. The red and green photoluminescences (PLs), even the blue one, are generated from the fluorescent layer, which absorb and down-convert the short-wavelength light emitting from chips lying beneath [3,4,5]. Although the former seems more straightforward than the latter, it requires a mass transfer printing technique which has not yet been reliable, judging from its current technical level. When using the method of manufacturing color conversion layer, the fluorescent layer is covered on the chips, and generates prime colors in situ. Therefore, it avoids the proved transfer printing technique, and is also able to realize chip matrices with very high resolution. This is the unique full-color solution for virtual reality and augment reality [6]. The bottleneck of the color conversion lies in the lack of fluorescent materials of high qualities. Being the color converter of a display is different to that of a general light source in the following four criteria. Firstly, the size of the grain (particle) should fall at the sub-micron scale, in order to couple the tiny-size mesa, which is expected to shrink to a few microns. Secondly, also due to the small dimension, the conversion efficiency, or more specifically, quantum yield (QY) and the absorption rate of the fluorescent materials, should be as high as possible to produce sufficient amount of long-wavelength light out of the short-wavelength light that it absorbs from the chip. Thirdly, in order to generate wide color gamut, the spectrum of the fluorescent layer should be as narrow as possible to obtain the high purity of each primary color without reducing the current efficiency [7]. Fourthly, the high refreshing rate of a cellphone screen when presenting moving objects [8], plus the potential application on visible light communication [9,10], requires a very short fluorescent lifetime. These four principles immediately rule out the traditional rare-earth doped yttrium aluminium garnet phosphor which has dominated the area general lighting. Semiconductor materials, such as CdSe/ZnS core-shell quantum dots (QDs), metal halide perovskite (MHP) nanocrystal (NC) or QD, have proved to meet all the above-mentioned criteria in general, and thus have received intensity investigations. Both CdSe and MHP QDs/NCs have partly been integrated with the liquid crystal display (LCD), serving as the back-light source, which achieve 100% and 123% National Television Systems Committee (NTSC) gamut coverage [11,12]. In comparison, the micro-LED display proves more energy-saving as it requires no backlight. In micro-LED display, these fluorescent QDs/NCs function as the color converter.

**Figure 1:**
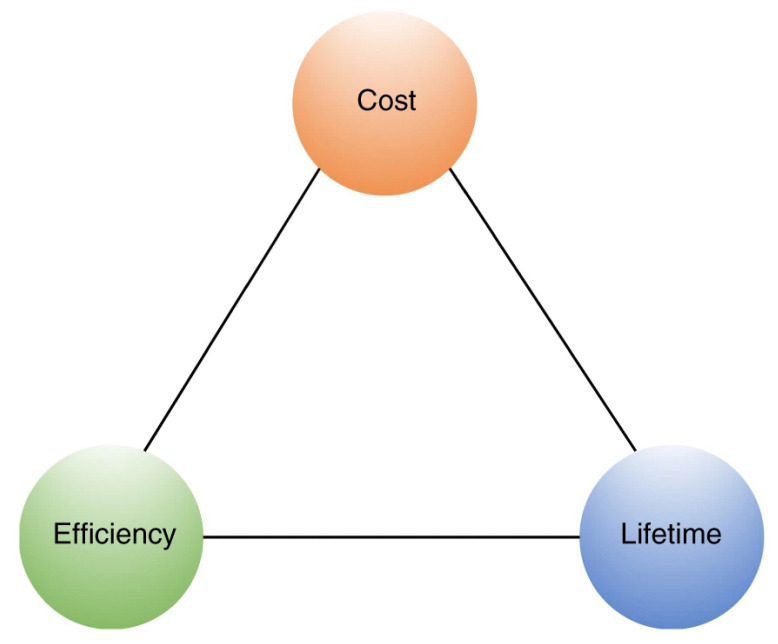
The model of golden triangle also applies for fluorescent material of micro-LED display. Reproduced from [13].

To evaluate the technical feasibility of a pre-commercialized optoelectronic material/device, there is a “Golden Triangle” model, presented in 2018 by Meng et al., who claimed that the high efficiency, low cost, and long lifetime are three equally important properties that a promising candidate should acquire before being successfully commercialized (Figure 1) [13]. Although this model was first established for solar cell, it also applies for the fluorescent material of micro-LED display.

**Figure 2:**
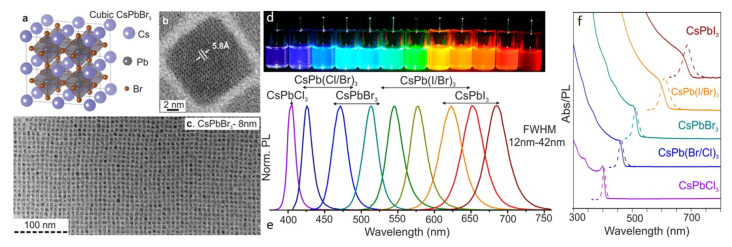
(**a**) The crystalline structure of CsPbX_3_; (**b**) The HRTEM image of a CsPbX_3_ NC; (**c**) TEM image of a CsPbX_3_ film; (**d**) image of CsPbX_3_ suspentions with different halide components under UV light, (**e**) the corresponding spectra, and (**f**) the XRD data. Adapted from [14].

The CdSe-based QD is currently more popular than MHP-QDs, but the some intrinsic characteristics prevent it from further development. The quantum confinement effect is adopted in CdSe-based QD to adjust the band-gap and consequently tune the color. As a result, the emission spectrum is highly sensitive to the particle dimension, e.g., the narrow spectral distribution, which is critical in display with high gamut, depends on a uniform size distribution. This requires strict temperature and time control in the synthesis procedure, and introduces extra cost in manufacturing. These problems can be avoided in MHP-NCs because of the different color/bandgap adjustment mechanism. The chemical formula of MHP is ABX_3_ (X = Cl, Br, and I, or a mixture of them), and the crystal structure and transmission electron microscopy (TEM) is illustrated in Figure 2a–c. By adjusting the halide elements and respective composition, the band-gap and color of the MHP can be tuned continually from near infrared to violet region, covering all the visible spectral region. The images, emission spectra and the absorption spectra are illustrated in Figure 2d–f, respectively. Compared to the size-dependent quantum confinement mechanism, the stoichiometric ratio of halides is easy to control. This sample is named as MHP-NC in this review.

In the form of NC, besides functioning as the color conversion layer of micro-LED display, it has been introduced to the LED for general lighting [15], as well as the scintillator for the X-ray detection [16]. The bottleneck for the MHP-NC lies in the long-term stability. It degrades rapidly when exposed to the ambient, as the QY and the spectra changes over time. Four main external factors in the ambient affect the stability: humidity (water), oxygen, thermal radiation and light irradiation. They attack the MHP-NC by altering the structure [17], in different manners respectively. These four factors coexist in the ambient, thus their effects are hard to be distinguished from each other. A majority of crystal structural alteration are negative in terms of the instant optical performance, but a minority of them are positive. Therefore, the first step towards enhancing the stability is to investigate the unique degradation mechanism upon each factor separately, whereby the combined pathway can be demonstrated in the second step.

A significant amount of work has so far been contributing to the mechanisms of degradation and to the solution to improve the stability of MHP-NCs. They roughly fall into four categories according to the main external factor involved. This article reviews the degradation mechanism of MHP-NCs upon each external factor, and the current progress of the application on the micro-LED display. It is organized as follows: firstly the degradation mechanisms induced separately by oxygen, water, thermal radiation and light irradiation, respectively; Secondly, the degradation pathway under multiple external factors which mimics the working environment of micro-LED display is analyzed; Finally, recent applications on the integration between MHP-NCs and micro-LED displays are also summarized.

## 2. Effects of Oxygen

There exist two main ways by which the oxygen atoms interact with MHP-NCs—oxidation and defect passivation. They exert opposite effects upon the optical performance, the former causes the PL quenching while the latter boosts the conversion efficiency.

**Figure 3:**
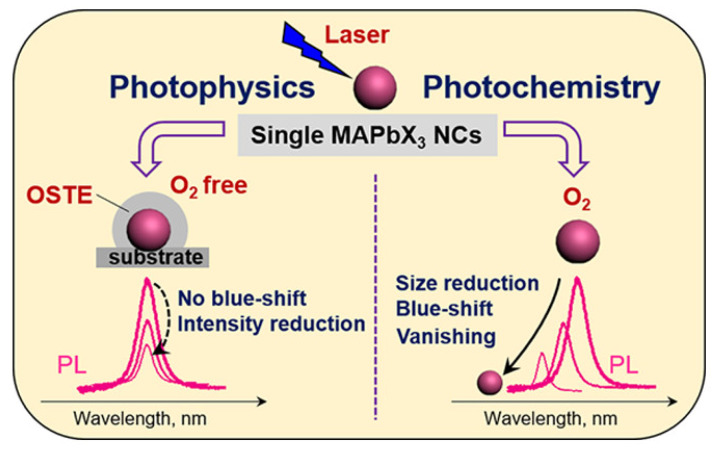
The degradation pathway in organic-inorganic hybrid MHP-NCs. Adpated from [20].

**Oxidation Effect**. The oxidation occurs when electrons have been captured by oxygen molecules. Haque et al. outlined the process that photo-excited electrons MAPbX_3_ NCs react with oxygen molecules to form superoxide O_2_^-^, which further react with CH_3_NH_3_^+^ to form volatile CH_3_NH_2_ [18]. Then, due to the removal of CH_3_NH_3_^+^ from the crystal lattice, the MAPbBr_3_ framework gradually decomposes. Scheblykin et al. further revealed that the structural collapse is mainly attributed to the migration of methyl ammonium ions (MA^+^) [19]. The destruction of the crystal structure also results in a blue shift of the PL peak position due to the NC size reduction and eventually leads to the PL vanishing. This degradation pathway in organic-inorganic hybrid MHP-NCs caused by “oxygen-assisted photoetching” is shown in Figure 3 [20].

**Figure 4:**
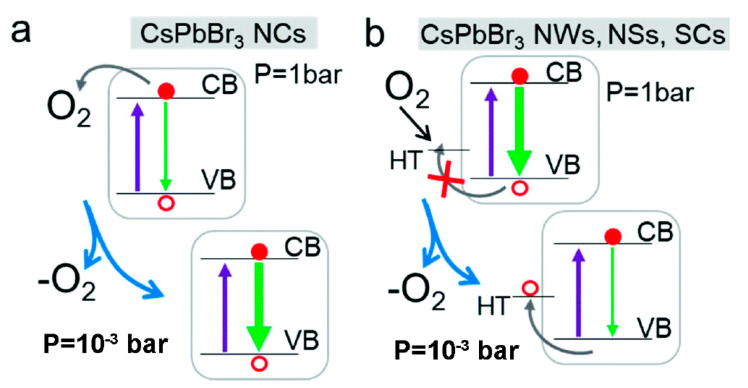
(**a**) Schematics of the interaction mechanism between O_2_ and CsPbBr_3_ NCs: oxygen removes photo-generated electrons (red circle) directly from the conduction band (CB) reducing the intensity (green arrow). Removal of O_2_ leads to enhanced efficiency. (**b**) Schematic depiction of the interaction between O_2_ and CsPbBr_3_ NWs, NSs and SCs: O_2_ coordinates electron-rich surface defects acting as non-radiative hole traps (HT). Removal of O_2_ results in lower efficiency with respect to atmospheric conditions. In ‘a’ and ‘b’ the excitation light is indicated by the pure arrows. Adapted from [22].

The exposure of MHP-NCs to O_2_ causes the direct extraction of photogenerated electrons. This is a direct competition with radiative exciton recombination, leading to a darkened PL efficiency under atmospheric conditions (Figure 4a). By performing the PL measurements in controlled atmosphere, Brovelli et al. show that the strong PL quenching is ascribed to the collisional interaction between MHP-NCs and O_2_. This PL quenching indicates that O_2_ can be used as a scavenger for photo-excited electrons without being affected by the structure defects [21]. The quenching of PL induced by O_2_ is a dynamic process, which competes with radiating exciton attenuation on a considerable time scale, rather than a static ultrafast mechanism [22]. Hence, this mechanism reduces the PL intensity at early stage, then keeping the attenuation dynamics basically unchanged afterwards.

**Defect Passivation**. It has been reported in literature that oxygen can promote the crystallization of MHP-NCs, as oxygen molecules can reduce defects at the crystal interface or inside the crystal [23,24,25]. Zeng et al. demonstrated that due to the “oxygen-boost” effect, oxygen physisorption on the surface of perovskite can change the electronic structure and repair trap states, enhancing PL intensity [26]. In higher-dimensional systems such as MHP nanowires (NWs), nanosheets (NSs) and single bulk crystals (SCs), the presence of O_2_ makes the surface hole traps passivated, enhancing the intensity, and the recombination kinetics remains unchanged (Figure 4b).

The two sorts of interactions post different effects on the optical performance, but they do not compete with each other. The defect passivation occurs within a limited time period from the moment MHP-NCs attach the oxygen, while the oxidation effect lasts and causes the constant quenching. Therefore, though the former can be harnessed for improving the quality of MHP-NCs, the oxygen molecules should be prevented from attaching or penestrating the MHP-NC. The development of packaging technology that serves as a strong oxygen barrier can provide better protection for MHP-NCs and further improve its stability. For instance, modifying the surface of MHP-NCs can greatly improve their stability to oxygen [27]. Strong steric hindrance of branched ligands in perovskite structure also can prevent O_2_ attacking. In future studies on stability, researchers will explore the use of ligands with short carbon chains on the surface of MHP-NCs with strong electron withdrawing groups and large steric hindrances.

## 3. Effects of H_2_O

The H_2_O, either in the phase of liquid or steam in the atmosphere, is another key factor that can alter the crystal structure of MHP-NCs. Similar to the effect of oxygen, the water posts both positive and negative effects upon the MHP-NC. Which one is dominant depends on the amount of the water presented, as well as the halide contents in the MHP-NCs.

**Figure 5:**
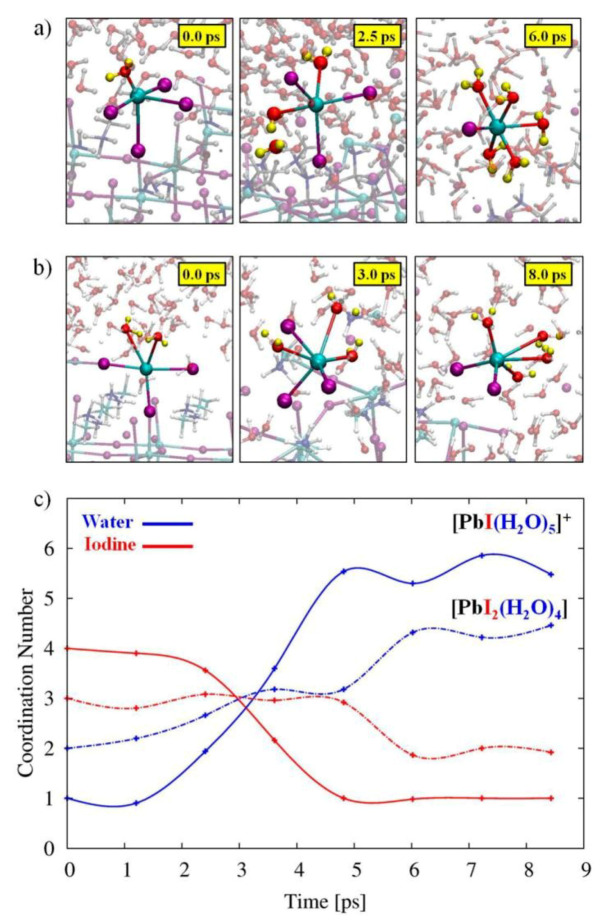
Representative geometrical structures of the formation of (**a**) [PbI_2_(H_2_O)_4_] and (**b**) [PbI(H_2_O)_5_]^+^ complexes. (**c**) Evolution of the Pb–I (red) and Pb–H2O (blue) coordination numbers for the formation of [PbI_2_(H_2_O)_4_] (dot-dashed lines) and [PbI(H_2_O)_5_]^+^ (solid lines) species. Adapted from [30].

**Negative: surface defects and surface recombination**. On one hand, Because the perovskite NCs are ionic compounds, the perovskite structure is easily degraded in water or even in a humid environment. Due to moisture induction, surface atoms fall off to cause surface defects, and it is also easy to cause agglomeration, which ultimately reduces quantum yield, affecting the luminous performance [28,29,30]. The degradation and agglomeration of perovskite NCs produce large grains, reducing the performance of the devices they constitute. Moreover, under light conditions, the effect of degradation will be further aggravated by the phenomenon of surface reorganization of NCs [17]. To study the possible effect of surface defects on determining the stability of the water/perovskite interface, the Angelis team simulated a PbI_2_ defect plate, which may be stable under PbI_2_ depleted conditions [30]. To this end, they adopted a surface terminated with PbI_2_, which seemed very inert in its clean form, and they removed six PbI_2_ units, leaving two undercoordinated Pb atoms on the exposed surface. Examination of the data in Figure 5 clearly shows that this defective surface has undergone a rapid degradation process. In particular, two insufficiently coordinated Pb atoms quickly desorb from the perovskite surface, resulting in the formation of solvated substances, shown in Figure 5a,b. One Pb atom is initially fixed to the surface of the perovskite by four I atoms, and a water molecule is added to the coordination sphere, shown in Figure 5a. The other surface Pb atom is initially bonded to three I atoms on the perovskite surface and two interface water molecules, shown in Figure 5b. During the CPMD trajectory, the Pb atoms on both surfaces deviate from the perovskite surface and form octahedral [PbI_2_(H_2_O)_4_] and [PbI(H_2_O)_5_]^+^ complexes in solution.

**Figure 6:**
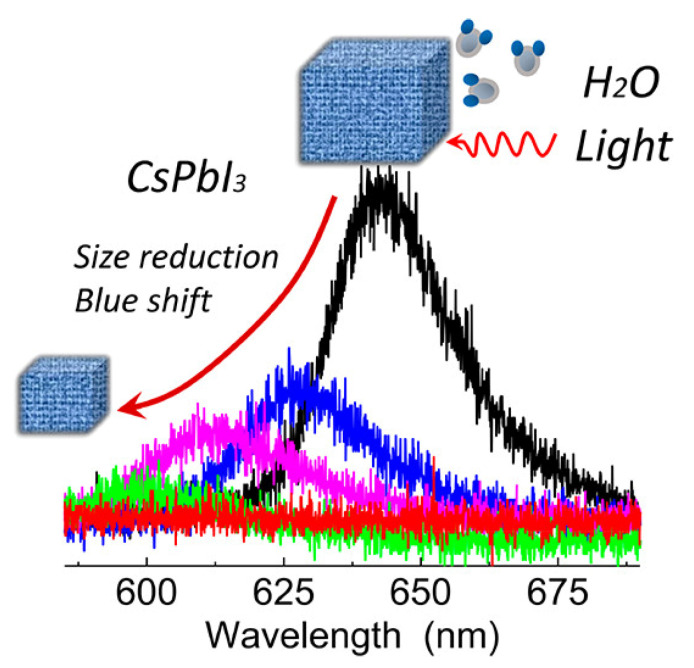
The water steam in the air dissolves the CsPbI_3_ NCs, and the size reduction causes fluorescence decrease as well as blue shift. Reproduced from [31].

The presence of large amount of water decompose the ionic-like MHP-NCs. Such decomposition is nonetheless rarely mentioned in experiments and theories. Yuan et al. monitored a significant fluorescence decrease under controlled atmospheric conditions (dry nitrogen, wet nitrogen, dry oxygen, and wet oxygen) for a single MHP-NC. They came to the conclusion that it is not oxygen but moisture alone that responsible for the fast degradation of CsPbI_3_ NCs in the dark—the dissociation of NCs can occur solely upon water steam. The stepwise (layer-by-layer) decomposition also induces the blue shift of the emission spectrum, as illustrated in Figure 6 [31].

**Figure 7:**
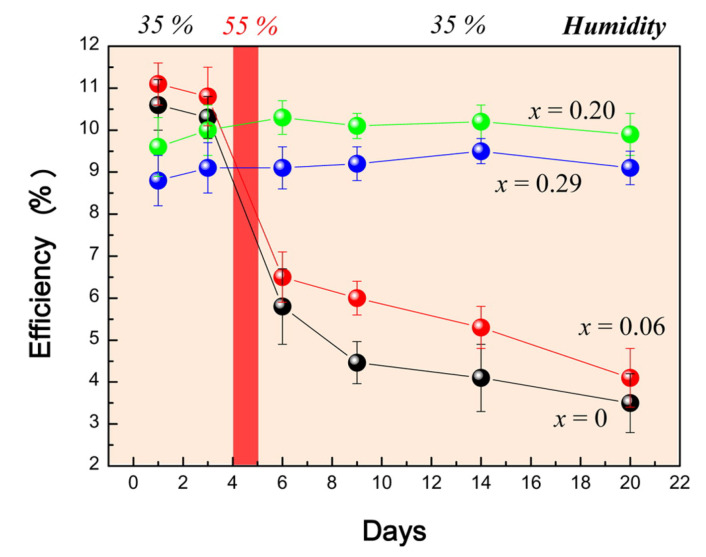
Power conversion efficiency variation with storage time, The sample is the heterojunction solar cells based on MAPb(I_1-x_Br_x_)_3_ (x=0,0.06,0.20,0.29), without encapsulation. The storage environment is air with Rh = 35% at room temperature. The samples are exposed to a RH of 55% for one day every fourth day to investigate performance variation at high RH. Adapted from [32].

Previous studies have found that CH_3_NH_3_PbI_3_ NCs decomposes when the relative humidity (RH) exceeds 55%, but it does not apply for CH_3_NH_3_PbBr_3_ NCs. The CH_3_NH_3_PbI_3_ NCs has a twisted cubic phase (tetragonal phase) when exposed to environment with RH > 55%, which means that CH_3_NH_3_PbBr_3_ NCs proves more stable than CH_3_NH_3_PbI_3_ NCs, as illustrated in Figure 7 [32].

**Figure 8:**
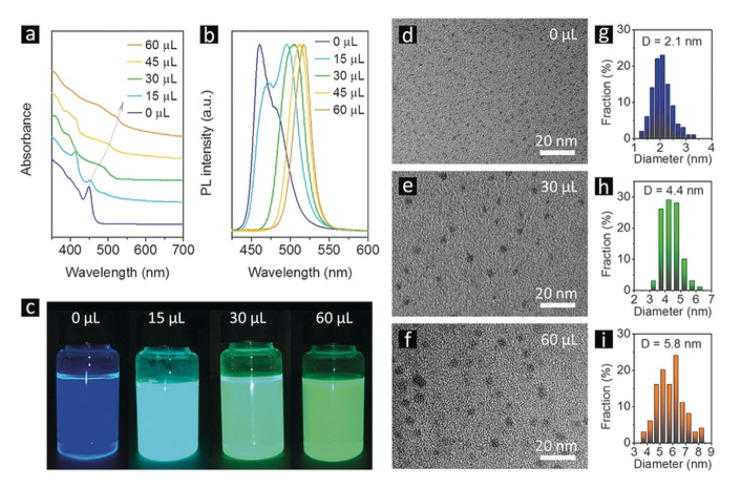
(**a**) UV/Vis absorption and (**b**) PL spectra of CsPbBr_3_ NCs synthesized using dry DMF and different minor amounts of water added into toluene, as indicated on the frames. (**c**) Photographs of CsPbBr_3_ NC solutions under UV light. TEM images (**d**–**f**) of CsPbBr_3_ NCs synthesized with 0, 30, and 60 upμL water additive, respectively. Adapted from [35]. (**g**–**i**) The size distributions of CsPbBr3 NCs synthesized with 0, 30, and 60 *upµ*L water additive, respectively.

**Figure 9:**
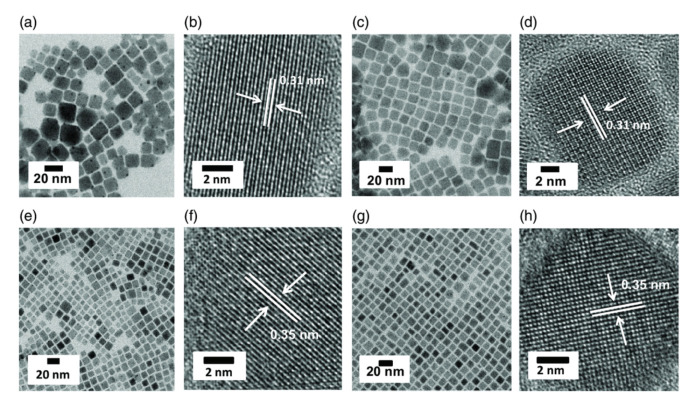
TEM and corresponding HRTEM images obtained for CsPbI_3_ NCs synthesized at (**a**,**b**) 0% RH and (**c**,**d**) 30% RH conditions. TEM and corresponding HRTEM images obtained for CsPbBr_3_ NCs synthesized at (**e**,**f**) 0% RH and (**g**,**h**) 30% RH conditions. Adapted from [36].

**Positive: Conducive to crystallization and reduce defects**. On the contrary, a trace of water would reinforce the crystallinity of the MHP-NC, therefore, reduce the surface defects (resembling the annealing process [33]) and increase the grain size [34]. As a polar solvent, water in the form of moisture can dissolve and remove excess ions and atoms present on the surface of CsPbX_3_ NCs, leaving the ideal unit cell.This water-annealing effect has been exploited in the synthesis process to enhance the quality of MHP-NCs. Zheng’s group reported that H_2_O molecules can be uniformly mixed with the precursor, resulting in a uniform water-assisted reaction after the precursor is injected into dry toluene, where uniformly distributed MHP-NCs are formed. The optical properties as well as the TEM images are illustrated in Figure 8 [35]. The mechanisms can be explained as follows. The tolerance coefficient of CsPbBr_3_ is 0.81, which is in the largest deviation from the ideal cubic structure. In fact, CsPbBr_3_ adopts an orthorhombic crystalline structure at room temperature. Since water molecules can coordinate with Cs^+^ ions, it is assumed that Cs^+^-*x*H_2_O (x>=1) coordination pairs (radius greater than the radius of the individual Cs^+^ ions) can act as cations during the formation of cubic CsPbBr_3_, so the cubic phase benefits large tolerance coefficient. Mamgain et al. found that CsPbX_3_ NCs synthesized under optimal humidity have higher stability and significantly improved optical properties. The explanation for this can be summarized as that water vapor can help repair common surface defects on CsPbX_3_ NCs, reduce non-radiative paths, and leave almost perfect CsPbX_3_ unit cells in solution [36]. In addition, the coordination pair of cation−xH_2_O disappears during purification. It was found that the CsPbBr_3_ NCs synthesized under the condition of 30% RH showed very strong green light under ultraviolet illumination compared with the CsPbBr_3_ NC prepared under the condition of 0% RH. NCs becomes more stable and shows enhanced optical performance [36]. In Figure 9, it is shown that the CsPbI_3_ NCs synthesized at 0% RH have a cubic shape with an average size of 13.6±2.7 nm. However, NCs synthesized in the presence of moisture are of cubic crystal shape, which have a uniform size distribution around 12.1±1.8 nm. CsPbBr_3_ NCs synthesized under the conditions of 0% and 30% RH are cubic, with uniform size distribution, and the average size is close to 9.9±1.8 and 9.3±1.7 nm, respectively.

**Figure 10:**
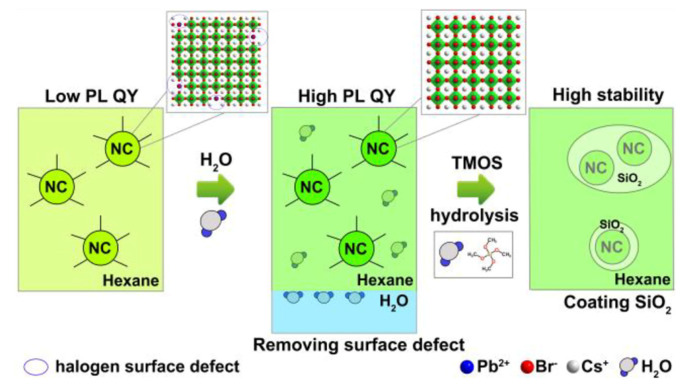
Illustration of the Water Treatment Procedure of CsPbBr_3_ NCs. Adapted from [38].

Water-assisted room-temperature synthesis of CsPbBr_3_/SiO_2_ nanocomposites with high stability also has been reported [37]. In the ethanol/water solvent system, the obtained nanocomposite has better optical characteristics and stability than the bare CsPbBr_3_ nanoparticles, and the prepared CsPbBr_3_/SiO_2_ nanocomposite can maintain 70% photoluminescence intensity even within 168 h [37]. In Liu et al.’s work, a trace amount of residual water facilitates the hydrolysis of tetramethyl orthosilicate, leading to the formation of SiO_2_-coated CsPbX_3_ NCs with high stability [38]. As shown in Figure 10, after water treatment, the surface defect layer of CsPbX_3_ NCs can be dissolved in water. Water is then removed from hexane solution, into which tetramethyl orthosilicate (TMOS) is added. A trace amount of residual water in the hexane hydrolyzed TMOS and the SiO_2_-coated CsPbX_3_ NCs can be obtained, which enhances PL intensity, and isolates further reactions from the outside environment and consequently enhances the stability.

**Figure 11:**
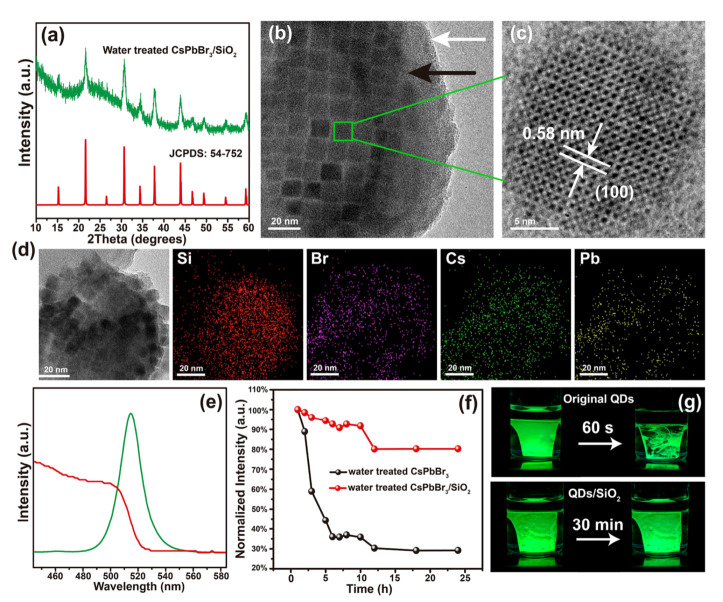
(**a**) XRD pattern of as-prepared CsPbBr_3_/SiO_2_ and the standard pattern. (**b**) TEM and (**c**) high-resolution TEM images of CsPbBr_3_/SiO_2_. (**d**) EDS elementary mapping. (**e**) PL and absoption spectrum. (**f**) PL intensity quenching with time period in the comparison between CsPbBr_3_ and CsPbBr_3_/SiO_2_. (**g**) The images of the quenching. Adapted from [38].

They proposed the reaction mechanism as an interface dissolution process. The reactions occur at the interface between water and hexane, which is immersible with water through ion movement due to the local structural instability of the NCs surface and the strong ion characteristics of CsPbBr_3_ perovskite. In addition, hexane has low solubility in water, as illustrated in Figure 11 [38].

It is also reported that there exists other possible reaction pathways, e.g., the formation of the hydrated compound Cs_4_PbBr_6_·2H_2_O after dehydration, during which water molecules are released by MHP-NCs without changing the lattice structure [39]. In summary, water treatment has been considered to be an important feature for future applications to improve optical properties in the hydrolysis reaction of MHP-NCs.

## 4. Effect of Light

Besides chemical reactions induced by the oxygen and H_2_O in the atmosphere, the light illumination also causes the structural transformation in MHP-NCs. The pathways of photodegradation which have so far been identified fall into three categories: (**a**) Photo-enhanced oxidation, which has been mentioned in the Section 2, that the light illumination reinforces the oxidation by producing photo-excited electrons, which are then seized by oxygen atoms and in consequence destroy the NC structures. (**b**) Defect-assisted photodegradation, in which the point defects accommodate the photoexcited electrons/holes, causing the deformation in lattices.

**Photo-enhanced oxidation**. The organic-inorganic MHP proves to be most vulnerable to the photo-induced oxidation. The most acute degradation under light is from the organic-inorganic hybrid MHP-NCs [40]. Due to the hygroscopicity of MA^+^, MAPbI_3_ is decomposed into CH_3_NH_2_, PbI_2_ and HI in humid environment. This decomposition process can be accelerated under light illumination [41]. Photoinduced production of superoxide O_2_^-^ can cause MAPbI_3_ to decompose into CH_3_NH_2_, PbI_2_ and I_2_.

**Figure 12:**
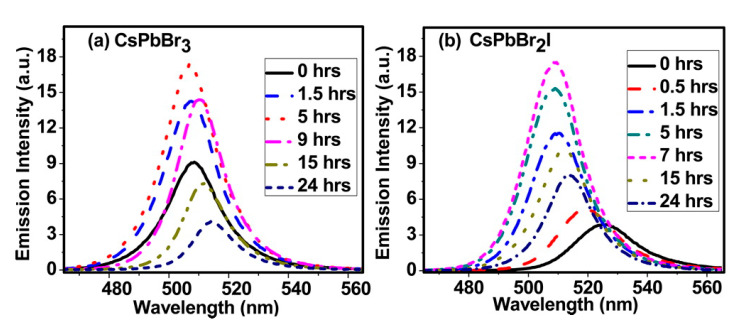
Emission spectra of (**a**) CsPbBr_3_ and (**b**) CsPbBr_2_I recorded following irradiation of samples for different periods. Reproduced from [42].

**Figure 13:**
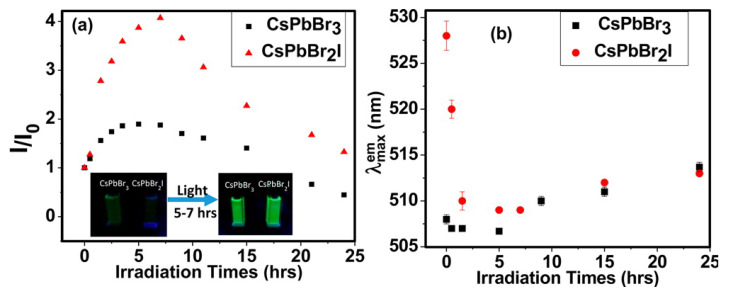
Plot of (**a**) relative emission intensity and (**b**) emission peak wavelength of the NCs as a function of light irradiation time. Inset of panel a shows the photographs of emission enhancement on photoactivation. Reproduced from [42].

**Defect-assisted photodegradation**. As mentioned in Figure 12 and Figure 13 [42]. Under continuous illumination, the fluorescence of NCs first experiences a transient enhancement, then decreases slowly, while the PL emission exhibits blue-shift initially and red-shift thereafter. This process is partially reversible. The transient enhancement of intensity is called “light activation”, which may be the result of the filling of some intermediate trap states by photogenerated charges. Sudipta et al. further speculated that the photoactivation can be ascribed to the photo-induced structural recombination. In the case of CsPbBr_2_I, it is possible to eliminate the iodine induced traps in distorted cubic crystals.

**Figure 14:**
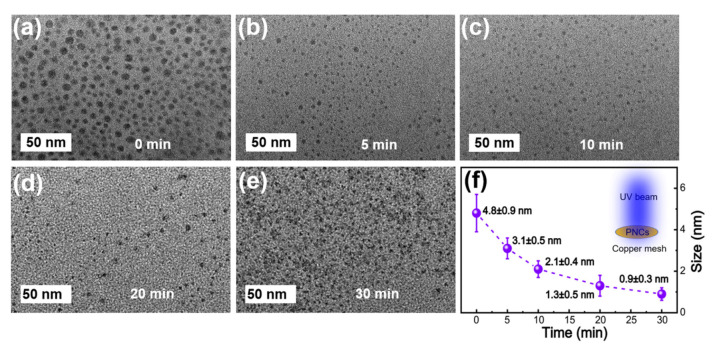
TEM images of the green-MAPbBr_3_ NCs exposed to 405 nm laser light for different amounts of time: (**a**) 0, (**b**) 5, (**c**) 10, (**d**) 20, and (**e**) 30 min. (**f**) Plot of the size versus time, revealing PNC size reduction with prolonging laser exposure time. Reproduced from [20].

Liu et al. further studied the blue-shift accompanied by the intensity enhancement. Figure 14a–e shows TEM images of green NCs after different laser exposure times. It can be seen that with the increasing exposure time, the average size of NCs decreases from 4.8 ± 0.9 to 1.3 ± 0.5 nm after 20 min, and finally decreases to 0.9 ± 0.3 nm after 30 min. The significant size reductions results in strong quantum confinement effect, which is responsible for the blue-shift.

**Figure 15:**
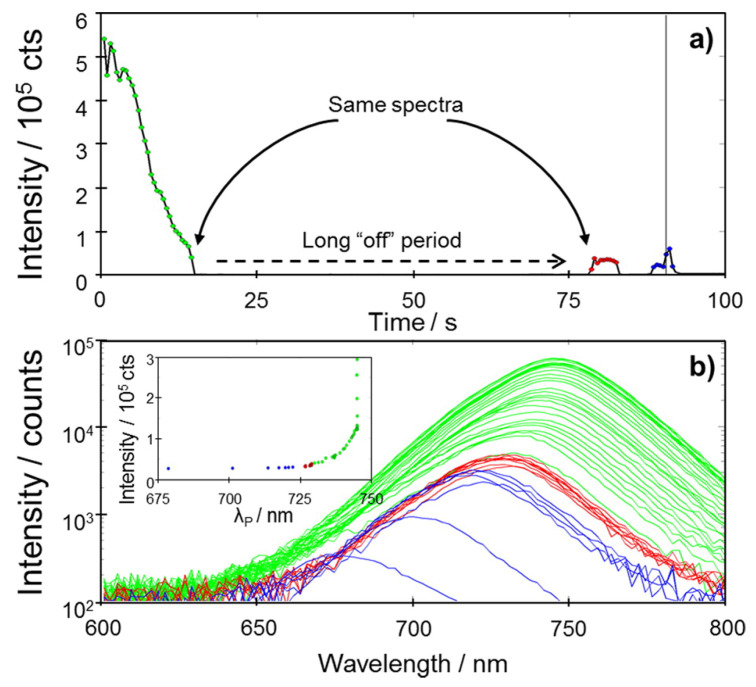
(**a**) PL intensity trace of a crystal showing degradation accompanied by blinking. The crystal then blinks on and off twice (marked with red and blue solid dots) after being in a dark state for >50 s. We also indicate with two black arrows that the spectra before and after the long “off” period are the same. (**b**) Spectral evolution of the crystal before (green lines) and after blinking (blue and red lines). The inset shows the correlation between peak wavelength and intensity despite the object being in “off” states for >50 s. Adapted from [19].

Aboma studied the photo-induced degradation of mapbi_3_ perovskite NCs under strong light excitation by using super-resolution fluorescence microspectroscopy. PL “blinking” was observed as is shown in Figure 15. The degradation process is temporarily terminated when the NCs are subjected to weak illumination, while exhibit degradation under strong illumination as more photogenerated charges enter those traps. It has been pointed out that the complete quenching of PL is the consequence of the fact that a majority of photogenerated charges access the photogenerated traps where they recombined non-radiatively.

**Figure 16:**
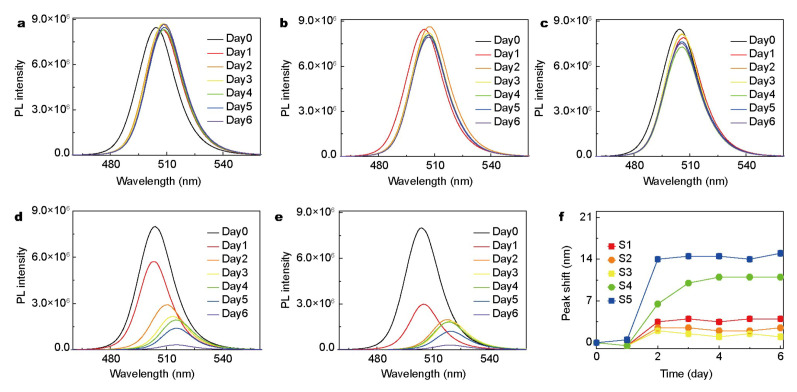
PL emission spectra of samples S1–S5 at different time. (**a**–**e**) are corresponding to samples S1–S5, respectively; (**f**) PL emission peak shift evolution over time for samples S1–S5. Adapted from [43].

**Figure 17:**
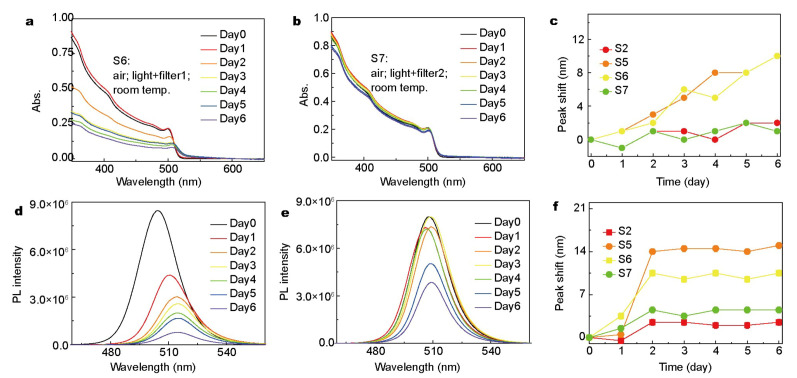
Absorption and PL emission spectra of samples S6 and S7 happen at different times during the test: (**a**) absorption spectra of sample S6 (with filter1:300–500 nm band pass filter), (**b**) absorption spectra of sample S7 (with filter2: 550 nm long pass filter), (**c**) evolution of 1st exciton absorption peaks for sample S2 and S5–S7, (**d**) PL emission spectra of sample S6, (**e**) PL emission spectra of sample S7, (**f**) evolution of PL emission peaks for sample S2 and S5–S7. Adapted from [43].

The crystals are exposed to light for the entire time, including the PL “dark” area. The degradation process is induced by photo-generated charges, rather than by environmental factors, such as high temperature. As shown in Figure 16 and Figure 17, lights of full visible spectral range and of 300–500 nm were used for illumination, respectively. In the latter case, not only the peaks in absorption and emission spectra red-shift, but also their intensities decrease. On the contrary, when the photon energy of the illumination light is lower than the band gap, only the decrease of photoluminescence intensity can be observed. The results show that the thermal effect plays a secondary role in the degradation of cspbbr_3_ and does not cause any structural damage [19]. They also pointed out that the trapped charge may be released from the trap states. This is a similar “blinking” phenomenon. In addition, degradation begins locally and then spreads throughout the crystal. The gradual change of the spectrum shows that the photo-induced charges recombine non-radiatively in the center of the trap and emit heat. Since the heat dissipation of QDs is limited, the excess heat alternates the structure of QDs. The relevant contents will be discussed in detail in the section of thermal stability.

**Figure 18:**
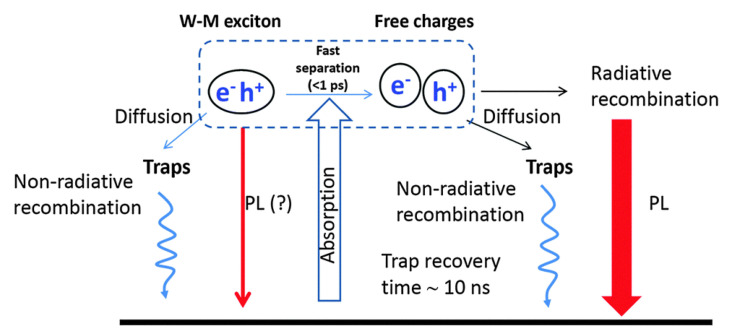
Scheme of the photophysical processes in perovskites. Initial absorption of a photon can create either Wannie–Mott exciton (exciton transition) or free electrons and holes (band to band transition). Due to the low binding energy, exciton splits to charges within a few picoseconds at room temperature. Excitons and free charges are highly mobile and can be trapped by trapping sites present at low concentration. The traps quench excitons and also induce non-radiative recombination of free carriers. Traps can become saturated (when concentration of the excitations is high) leading to suppression of quenching. The trap concentration can be changed by a photochemical reaction involving oxygen. Adapted from [44].

In short, photodegradation is an important factor affecting the stability of NCS. As shown in Figure 18, the initial absorption of photons can produce excitons, free electrons, and holes. Due to the low binding energy, the exciton splits into electron and hole rapidly at room temperature. Exciton and free charge have high mobility and can be trapped by low-concentration defect sites. The trap can quench the exciton and induce the non-radiative recombination of free carriers. Under very strong light illumination, the trap can be saturated and the quenching phenomenon can be suppressed [44].

**Figure 19:**
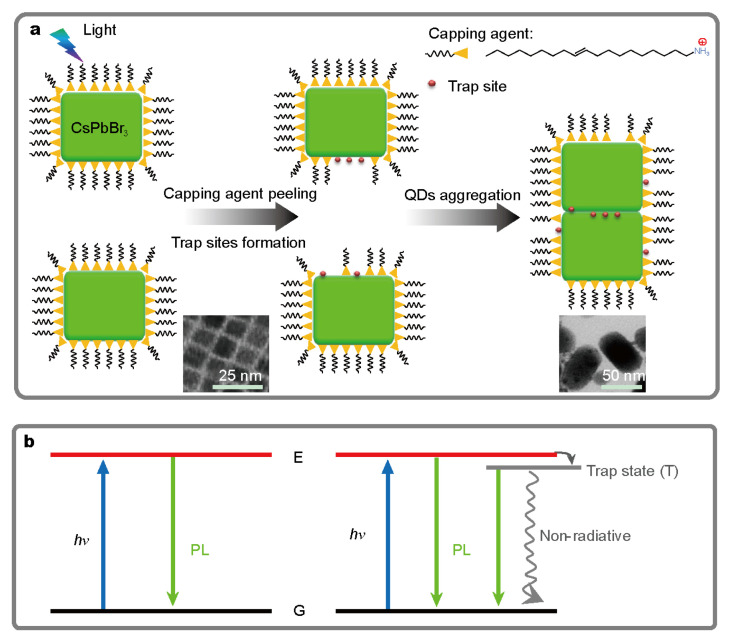
(**a**) Schematic picture of the photo-degradation pathway occurring on CsPbBr3 QDs in toluene. (**b**) Mechanism of the various excited-state decay pathways for CsPbBr3 QDs. Adapted from [43].

Due to the high carrier mobility and fast interface charge transfer of CsPbBr_3_ QDs, the charge generated by light excitation may diffuse to the surface, then can be captured by the ionic ligands. Because the ionic nature of the interaction between CsPbBr_3_ QDs and ligands=, their binding is very dynamic. The ligands are easily removed from the surface of QDs and dissolved in solvents. Without the protection of ligands, neighboring particles tend to aggregate and the size increases, resulting in red-shift of absorption and PL spectra. This process can be vividly shown in Figure 19. The optical excitation of charge is the key premise. By studying the dynamics of this process, it is proposed that light-induced aggregation of QDs would produce passive and non-emission trap states in quantum dots. This explains the decrease of QY and the prolongation of photoluminescence lifetime after photodegradation of QDs [43].

Xu et al. proposed that the disappearance of excitonic peak in the absorption spectrum indicates a quenching of band edge exciton, which is responsible for the PL deterioration of two MHP-NCs [45]. The excited carrier undergoes a thermal relaxation process and then drops to a lower energy level. It also means that more defects in MHP-NCs lead to irreversible decomposition process under light, which may be attributed to the local electric field caused by trapped charge. Based on the above findings, a theory of the influence of light on the degradation process of MHP-NCs can be explained as follows: in the process of preparing MHP-NCs, there are inevitably defects such as impurities, vacancy and hanging bond. Then these defects act as electron traps on the crystal surface. and capture photo generated carriers, thus forming a local trap state. Once exposed to light, the trap density increases, which disturbs the crystal structure and generates an electric field. In turn, it enhances the coupling between the carriers and the lattice, resulting in lattice distortion. The final result is MHP structure degradation.

It is necessary to point out the difference in degradation mechanisms between NCs and bulk perovskite. It has been well accepted that the specific surface area of NCs increasse as the size decreases. On one hand, the degradation of NCs are more likely to be accelerated compared with bulk perovskite, due to the larger number of surface defects; On the other hand, the ligands anchored on the surface of NCs block the migration channel of defect ions, alleviating the aging. Although there exist differences in the structure and properties between NCs and bulk, it is still meaningful to understand the degradation mechanism of bulk, such as quenching and intensity decay, which also occur in NCs. A large number of excitons and free charges are excited by long-term excitation. In the case of QDs, they are confined in each NCs, but move without such obstacles in the case of bulk. Huang et al. proposed that the MAPbBr_1.5_I_1.5_ MHP-NCs produce irreversible degradation in the effect of photoelectric field [40]. Light first induces the migration of halide ions and forms the enrichment areas containing MAPbBr_1.5−*x*_I_1.5+*x*_ (iodide rich) and MAPbBr_1.5−*x*_I_1.5+*x*_ (bromide rich) respectively. Furthermore, the iodide aggregation area further decomposes into PbI_2_, and finally degenerates to Pb^0^. The local iodine and bromine richness induce the red-shift and blue-shift in local absorption peak, respectively. Li’s group in University of Bayreuth identified the fact that it is the gradient of light illumination rather than the light illumination itself that drives the iodine migration. The area of CH_3_NH_3_PbI_3−*x*_Cl_*x*_ film that is subjected to excitation shows no PL reduction, while the boundary of the light soaking area exhibits a dark ring. There exists, as they proposed, an iodine concentration surrounding the beam area, resulted from the the uneven halide mobility at the edge of the beam area. The migrated iodine ions leads to interstitial defects which in consequence causes the PL reduction [46].

## 5. Thermal Effect

**Figure 20:**
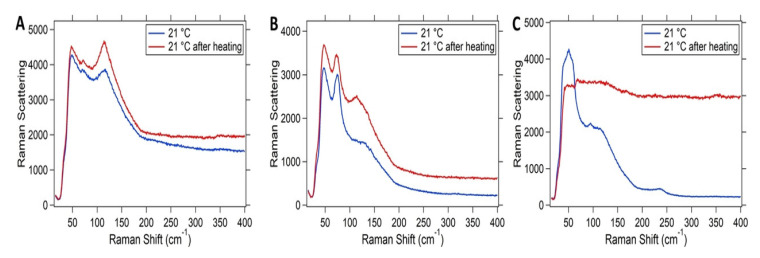
Raman spectra of (**A**) CsPbCl_3_, (**B**) CsPbBr_3_, and (**C**) CsPbI_3_ NCs before and after heating to 250 ${}^{\circ }$C and allowing the samples to cool. Reproduced from [47].

Boote et al. studied the thermal stability of perovskite NCs by heating the samples in a glove box [47]. There are three different results due to the increase of temperature for three kinds of halide perovskite NCs. The structure of CsPbI_3_ NCs changed from 50 ${}^{\circ }$C due to the combination of PbI_2_ and δ CsPbI_3_. At 250 ${}^{\circ }$C the phase transition is basically completed, and all the reflection of γ phase disappear. CsPbBr_3_ NCs show similar behavior to the measurement of optical stability when heated, i.e., the crystal growth results in the reduction of reflection width and the appearance of clear orthogonal pattern, and the crystal structure even lasts until 250 ${}^{\circ }$C. Both CsPbCl_3_ and CsPbBr_3_ samples exhibited crystal growth under irradiation or heating. Under the same conditions, the large CsPbI_3_ crystal (34±5 nm) is the most unstable component, the XRD reflection and Raman band decrease under irradiation, and the γ-phase returns to the non luminescent δ phase after heating. The smaller CsPbI_3_ NC (14±2 nm) purified by different washing methods showed good optical stability without crystal growth signs, but it was still thermally unstable. Dastidar et al. [48] found that CsPbI_3_ was stable in the cubic phase when the temperature was below 100 ${}^{\circ }$C in the dry environment, and partial degradation was observed at 100 ${}^{\circ }$C in the environmental measurement, while no degradation was observed in the sample without water. Therefore, water does not change the degradation process, but may change the temperature at which degradation begins. After a heating cycle, the Raman spectra of each halide component were collected. As shown in Figure 20, CsPbI_3_ is the only sample that fails to recover the original band structure [47]. The effect of temperature on the luminescent band of CsPbX_3_ NCs was observed. It is found that when the heat energy is close to the trap state energy, the temperature rise leads to the luminescence quenching [49]. By comparing the normalized spectrum with the slight shift of the peak wavelength, CsPbCl_3_ samples show a slight red shift with the increase of temperature. After cooling back to room temperature, the red shift seems to be partially reversible. For CsPbBr_3_ and CsPbCl_3_, a slight blue shift was observed during the heating process, but CsPbBr_3_ did not move back after cooling, indicating that an irreversible process occurred. In contrast, CsPbI_3_ completely restored the original maximum value of luminescence. Irreversible process usually involves the change of material interior, while reversible process involves the change of surface state.

**Figure 21:**
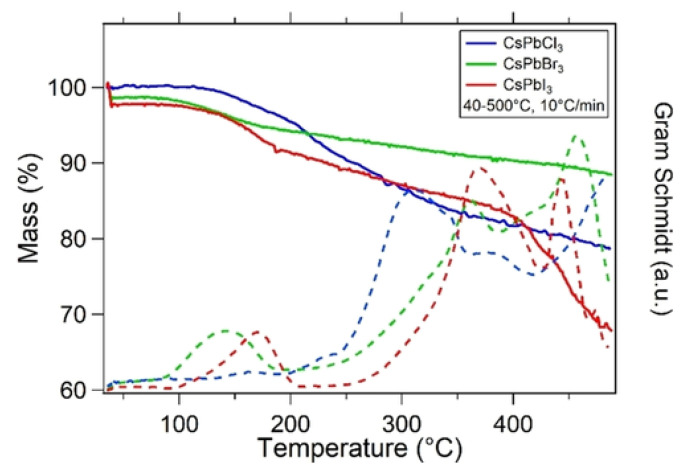
TGA-FTIR Mass loss and Gram-Schmidt curves for CsPbX_3_ NCs from 40–500 ${}^{\circ }$C. The Gram-Schmidt curves monitor the total change in FTIR signal over time, indicating FTIR detection of mass loss products. Reproduced from [47].

**Figure 22:**
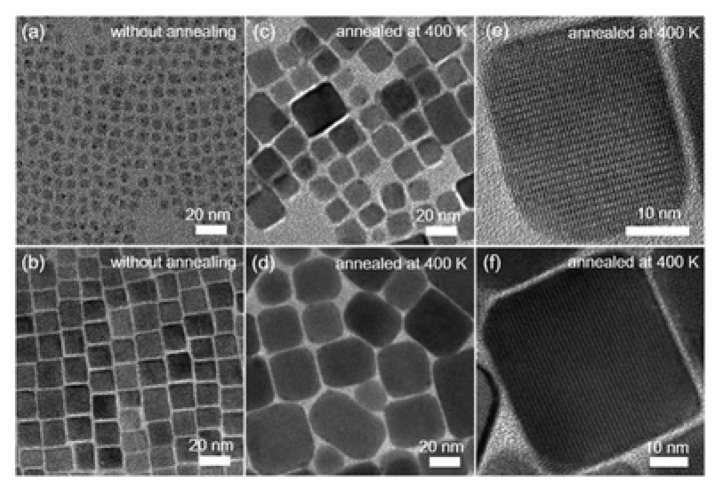
Low-and high-resolution TEM images of CsPbBr_3_ NC492 (**a**,**c**,**e**) and NC517 (**b**,**d**,**f**) at room temperature. (**a**,**b**) and (**c**–**f**) refer to TEM images of the CsPbBr_3_ NCs without any thermal annealing and with thermal annealing at 400 K for 20 min, respectively.Reproduced from [50].

The thermal decomposition products were further analyzed by Thermometric Analysis-Fourier Transform Infrared spectrometer (TGA-FTIR), and the results are shown in Figure 21. In addition to the mass loss, the gram schmidt curves for each sample are shown, which represent the total change in the FTIR signal based on continuous measurements. TGA-FTIR analysis show that the mass loss of NCs was only due to the release of surface ligands and subsequent degradation. The mass loss in the range of about 100–200 ${}^{\circ }$C in all samples corresponds to the surface ligands of oleic acid, while other mass loss above 250 ${}^{\circ }$C may be due to the CO_2_ generated by the reaction of surface ligands with O_2_ in the air stream. In all cases, no gas loss products containing cesium, lead or halides were detected. Furthermore, Yuan et al. separately heated and annealed the perovskite nanocrystalline samples. The typical TEM images of CsPbBr_3_ NCs without any thermal annealing and 400 K thermal annealing are shown in Figure 22. The NCs without heat treatment have cubic morphology, and the average cubic length of the two CsPbBr_3_ NCs are 5.5 and 13.7 nm, respectively. After annealing at 400 K for 20 min, the average size of CsPbBr_3_ NCs increased to 17.3 and 31.6 nm, respectively. As shown in Figure 22e,f, clear lattice fringes are observed in the high-resolution TEM image of the 400 K annealed sample, indicating that it has high crystallinity. The lattice fringes pass through the whole NC straight line, leaving no trace of the original 5.5 and 13.7 nm cubes, which shows that the growth of CsPbBr_3_ crystal can not be simply considered to be the result of the aggregation of small particles, but more likely due to the deeper physical process [50].

## 6. Combination Effect of Light and Ambient Conditions

**Figure 23:**
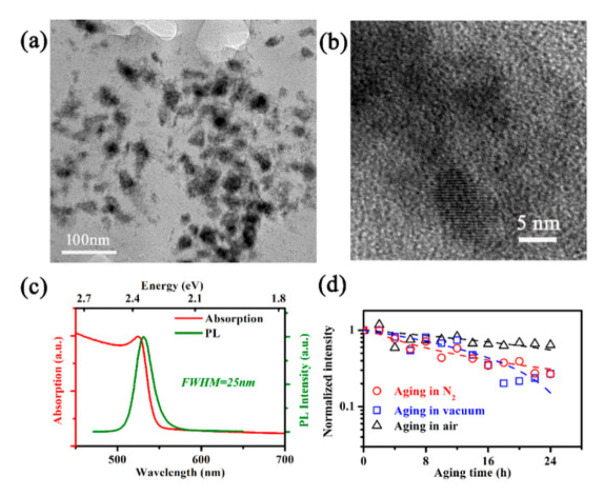
(**a**) A typical TEM image MAPbBr_3_ NC in PVDF matrix; (**b**) TEM image of a typical MAPbBr_3_ NC; (**c**) normalized absorption and PL spectrum of the fabricated MBNCs-PVDF; and (**d**) PL intensities recorded during aging, normalized to the initial intensity, and presented in logarithmic ordinate. Hollow circles correspond to aging in N_2_, hollow squares to aging in vacuum, and hollow triangles to aging in air, while dashed lines are obtained by linear fitting for aging in vacuum and air and by reciprocal fitting for aging in N_2_. The temperature and 368 nm UV light illumination density during aging are 323 K and 5.6 mW/cm${}^{2}$, respectively. Reproduced from [51].

When the MHP-NC is incorporated in the micro-LED display as the fluorescent layer, it is subjected to constant illumination of short-wavelength light from chips and ambient light as well. Despite the encapsulation’s prevention, the air penetration always exists, which brings in water steam and oxygen. In addition, the radiation from the chips, in conjunction with the heat, causes the temperature of the MHP-NC slightly higher than room temperature. These external factors exert a combined stress on the MHP-NC thus it should be taken into account as a whole when investigating the stability of MHP-NCs for fluorescent layer in micro-LED display. The work studies the stability of the green light MAPbBr_3_ NCs which are synthesized in situ and embedded in polyvinylidene fluoride or polyvinylidene difluoride (PVDF), namely MHP-NC-PVDF. Under the combined effect of 368 nm ultraviolet light irradiation at 323 K ambient temperature and power density of 5.6 mW/cm${}^{2}$, they were aged in nitrogen, vacuum and air atmospheres respectively, and the PL quenching characteristics of the synthesized NCs were studied.The average size of NCs used in the experiment is 15 nm (Figure 23a,b). The distribution of NCs in PVDF film has good oxygen resistance and water resistance, which is helpful to study the internal stability of NCs. At 300 K, the absorption spectrum (Figure 23c) shows an obvious absorption peak at 2.37 ev (524 nm), while at 2.34 ev (531 nm), the full width at half maximum(FWHM) of PL peak is 25 nm. In this work, the PL peak wavelength of NCs is shorter than that of bulk MAPbBr_3_ (545 nm). We then divided the original samples into three groups, aged in nitrogen, vacuum and air for 24 h, with the same stress. After vacuum agingand and N_2_ aging, the photoluminescence intensity of MHP-NC-PVDF decreased to 25% of the original sample, and the photoluminescence intensity of air aging sample was 64% (Figure 23d).

**Figure 24:**
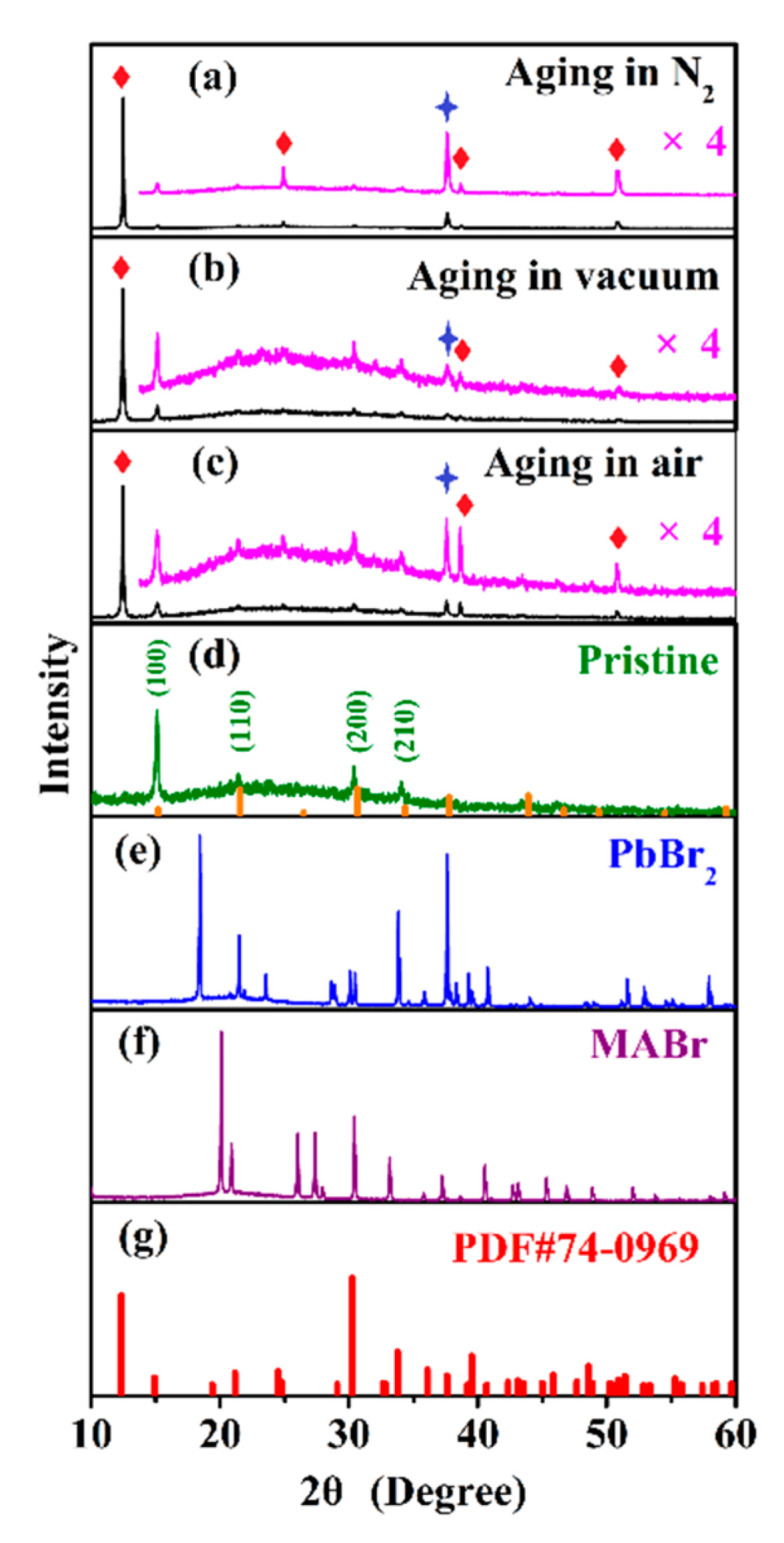
XRD results of MHP-NC-PVDF: (**a**) after aging in nitrogen atmosphere; (**b**) after aging in vacuum; (**c**) after aging in air atmosphere; (**d**) unaged MBNCs-PVDF, the bottom yellow line is Standard PDF card for MAPbBr_3_ (PDF # 54-0752). (**e**) XRD spectrum of PbBr_2_; (**f**) XRD spectrum of CH_3_NH_3_Br (MABr); (**g**) standard PDF card of NH_4_Pb_2_Br_5_ (PDF # 74-0969). The diamond in (**a**–**c**) indicates the diffraction peak of NH_4_Pb_2_Br_5_, and the star indicates the diffraction peak of PbBr_2_. Reproduced from [51].

According to the mechanism that light causes the C−N bond in CH_3_NH_3_^+^ to break [45,52], combined with the above XRD results Figure 24, the reaction process of NH_4_Pb_2_Br_5_ can be further sorted out. First, under the synergy of near ultraviolet light irradiation and heating, MAPbBr_3_ NCs decompose into NH_4_Br, PbBr_2_ and (−CH_2_−), namely:

(1) $${\mathrm{CH}}_{3}{\mathrm{NH}}_{3}{\mathrm{PbBr}}_{3}\;⟶\;{\mathrm{NH}}_{4}\mathrm{Br}+{\mathrm{PbBr}}_{2}+(-{\mathrm{CH}}_{2}-)$$

Then, NH_4_Br and PbBr_2_ further react to obtain NH_4_Pb_2_Br_5_, namely:

(2) $${\mathrm{NH}}_{4}\mathrm{Br}+2{\mathrm{PbBr}}_{2}\;⟶\;{\mathrm{NH}}_{4}{\mathrm{Pb}}_{2}{\mathrm{Br}}_{5}$$

To further distinguish the relative contribution of 323 K temperature environment and near-ultraviolet light irradiation to the decomposition, aging studies were carried out at room temperature near-ultraviolet irradiation and separate heating. It was proved that the aforementioned decomposition reaction was the result of the synergy between heating and near-ultraviolet irradiation. As the test temperature increases, the peak energy of the PL spectrum of MHP-NC-PVDF is blue-shifted. This blue shift phenomenon is consistent with the common characteristics of MAPbX_3_ materials [53]. When the test temperature is lower than 70 K, the PL spectrum of MHP-NC-PVDF has a clear low-energy band tail. This tailed emission is reported in the PL spectra of MAPbBr_3_ thin films and single crystals, which originates from the combined emission of excitons bound by the defect state [54]. As a brief summary, the working condition in micro-LEDs is hash for MHP-NCs, the simultaneous stresses of UV light, air penetration and slightly heating cause the decomposition of organic-inorganic hybrid MHP-NC. The MA^+^ organic ions prove the most vulnerable, thus, unsuitable for functioning as the color conversion layer. In the future, investigations on the stability of inorganc MHP-NCs should be carried on under the similar environment.

## 7. Measures to Improve the Stability of MHP-NCs

Under high humidity environment, the luminous performance of MHP-NCs is reduced, after exposure to light and heat radiation, which hinders its application in the field of Micro-LED display. Therefore, researchers have explored many strategic measures to improve the stability of MHP-NCs.Recent strategies to improve stability have focused on the regulation of MHP components, passivation of surface defects, ligand engineering and encapsulation [55,56]. The contribution of low dimensional PNC using to improve the stability is also involved.

**Component regulation**. By partially replacing the A-site [57] and B-site [58] in the inorganic perovskite NCs by cations, The method adjusts the composition of the perovskite NCs to improve its chemical stability. In Amgar’s work, the introduction of Rb^+^ ions into CsPbX_3_ system is proposed. The addition of a small amount of Rb^+^ ions affect the structural pressure level of inorganic CsPbX_3_(X=Cl,Br) perovskite core. It can be interpreted as the increase of octahedral tilt, which affects the reverse bond overlap of Pb^2+^ and X^-^ orbit. Finally, the mixed cation perovskite NCs are formed. Their properties are very similar to those of CsPbX_3_ NCs. These hybrid Rb^+^/Cs^+^ NCs are easier to adjust the structural distortion caused by cation substitution than their bulk NCs. The possibility of moderate band gap tuning of Rb^+^/Cs^+^ mixed cation NP is a step in the overall understanding of perovskite NCs. However, a new problem arises from this substitution, i.e., the black cubic(α-) phase of CsPbI_3_ is unstable at room temperature. A new strategy is discussed, i.e., to partially replace Pb2+ (B-cation) with different metal ions, as a possible way to improve the thermal stability and phase stability of ABX_3_ perovskite. The 6s and 6p orbitals of Pb2+ constitute the maxima of valence band and the minima of conduction band for CsPbX_3_ perovskite, respectively. Therefore, using other metal ions instead of Pb2+ for phase stabilization may result in deep defect states and destroy the defect tolerance of the host CsPbX_3_. These new defects may act as traps and scattering centers, which affect the photoelectric properties. So Swarnkar proposed that the effects of various B-site replacement on the crystal structure and electronic structure of CsPbX_3_ still need more detailed understanding.

**Figure 25:**
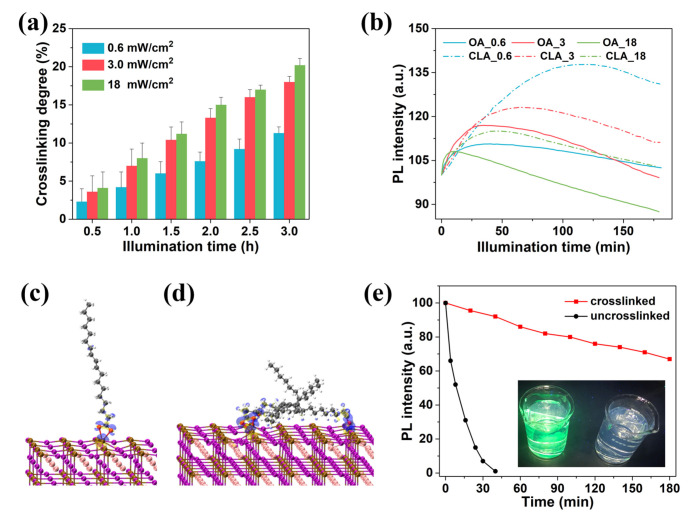
(**a**) Cross-linking degree of CsPbBr_3_-CLA QD films with varied illumination intensity and time. (**b**) PL intensity of CsPbBr_3_-OA and CsPbBr_3_-CLA QD films upon continuous illumination time. Surface charge redistributions of optimized PbBr_2_−rich CsPbBr_3_ surfaces with (**c**) CLA and (**d**) poly-CLA ligands’ modification. Electron density difference δρ(r): isosurfaces taken at 0.003 e Angstrom-3. Yellow means gaining an electron, blue means losing an electron. (**e**) The water resistance test of uncross-linked and cross-linked CsPbBr_3_-CLA QD films with a thickness about 500 nm. The inset is the photograph of uncross-linked and cross-linked CsPbBr_3_-CLA QD films after being immersed into water for 3 h upon UV light excitation [64].

**Ligand engineering**. In the synthesis of perovskite NCs, in order to improve the stability, long-chain organic ligand such as oleic acid and oleylamine are usually introduced to prevent the agglomeration of perovskite NCs to a certain extent, and it has a hydrophobic effect to stabilize the perovskite NCs [59,60]. Moreover, the use of amine-based passivating materials (APM) has been proposed and proven to be effective [61]. Yang has pointed out the role of amino and carboxyl groups in the repair of internal defects of MHP-NCs [62]. Dong’s group adopted a ligand-assisted SDM (LASDM) method, which can synthesize PNC in situ in a polymer matrix, with high stability, wide color adjustability and good color purity, and proved that it is a kind of suitable for various perovskite precursor compounds, various polymer substrates and different processes [63]. Wei et al. [64] have successfully realized the whole solution processing of PNC LED devices by in situ photo-initiated ligand crosslinking. Conjugated linoleic acid(CLA) used in the experiment has a long alkyl chain structure similar to the traditional oleic acid ligands, with carboxyl groups at the ends, which can effectively guarantee the colloidal stability of the NC it attached. In addition, CLA also has conjugated diene bond, which gives the material the property of crosslinking polymerization. Perovskite NCs can be used as photoinitiators to produce free radicals. Under illumination, CLA connect the neighboring NCs to form a cross-linked network structure. The cross-linked QD films are no longer dissolved in non-polar solvents, so the charge transfer layer can be processed in the upper layer of perovskite by solution processing. After crosslinking, the NCs still maintain their initial morphology and crystal structure, which is accompanied by the enhancement of fluorescence properties. Seeing that the traditional oleic acid ligands are easy to desorb on the surface, resulting in poor stability. However, the binding energy between CLA and perovskite is enhanced after crosslinking, which greatly improves the stability of NCs. After soaking the QD film in water for 3 h, its fluorescence remains more than 60% of the initial value. Moreover, its fluorescence still maintains 80% of the initial value after storing the crosslinked QD in air for one month, as is shown in Figure 25. The results of SEM and AFM show that the initial film morphology can still be effectively preserved when the charge transfer layer is processed in the upper layer of perovskite film with non-polar solvent, and the solution processed charge transfer layer still has an ideal film morphology. This method can be used to realize the whole solution processing of low cost and high efficiency optoelectronic devices.

**Figure 26:**
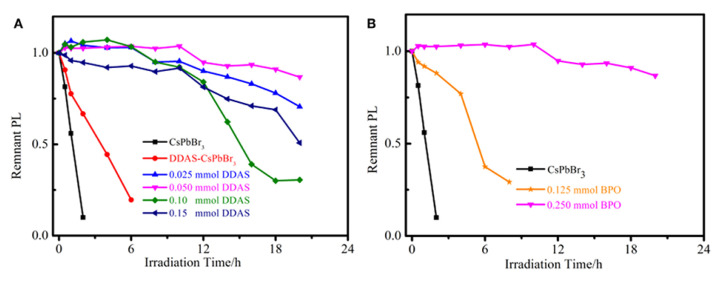
(**A**) Photostability of the CsPbBr_3_ NCs, DDAS–CsPbBr_3_ NCs (0.050 mmol DDAS treatment), and 0.250 mmol BPO oxidized DDAS–CsPbBr_3_ NCs with different DDAS treatment. (**B**) Photostability of the different BPO oxidized DDAS–CsPbBr_3_ NCs (0.050 mmol DDAS treatment). Reproduced from [68].

**Cladding by organic polymers or inorganics shells**. Encapsulation of perovskite NCs using organic polymer film or inorganic compound powder with high chemical stability as a matrix is another method to protect perovskite NCs and improve their stability. Moreover, this method is compatible with the traditional photoluminescence LED packaging process, and is more suitable for the field of photoluminescence LED. Song et al. encapsulated the CsPbBr_3_ perovskite NCs synthesized by the heat injection method into ethyl cellulose membranes. After 150 h of air stability testing, the perovskite NCs protected by ethyl cellulose membranes were luminous. The quantum yield drops by about 7.4%. This is because the hydrophobic ethoxy group in ethyl cellulose prevents the direct contact of perovskite NCs with water molecules to a certain extent [65]. Bit group [66,67] uses polyvinylidene fluoride to coat perovskite quantum dots. PVDF membrane has inherent excellent hydrophobicity, so even if it is immersed in water for 400 h, the composite membrane has almost no change. Due to the local electric field induced by the trapped charge along the grain boundary, reducing the defects in the perovskite is considered to be an effective way to improve its optical stability. Liu et al. used lead oxide to coat MHP-NCs through peroxide post-treatment [68]. First, CsPbBr_3_ NCs (DDAS-CsPbBr_3_) was modified with dodecyl dimethylammonium sulfide (DDAS), and then benzoyl peroxide (BPO) was used in turn. In this way, lead can be easily oxidized and lead oxide grows rapidly on the surface of the single CsPbBr_3_ NCs. Under the illumination of 450 nm LED lamp (175 mW/cm${}^{2}$), CsPbBr_3_ NCs coated with PbO remained at 90% of the initial PL intensity within 20 h, as shown in Figure 26. Dong’s group developed a micro-encapsulation strategy to achieve an organic-inorganic perovskite-polymer composite membrane with high luminous efficiency, color purity, and ultra-high stability to heat and water exposure [69].

**Figure 27:**
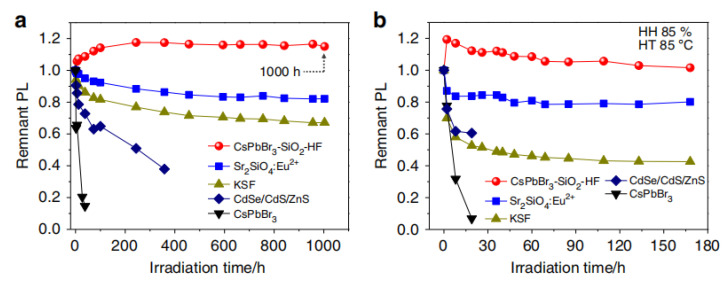
Photostability of the CsPbBr_3_–SiO_2_–HF. (**a**) Photostabilities of the CsPbBr_3_-SiO_2_-HF, ceramic Sr_2_SiO_4_:Eu2+ green phosphor, KSF red phosphor, colloidal CsPbBr_3_ NCs and CdSe/CdS/ZnS NCs under illumination, sealed with Norland-61 on the LED chips (20 mA, 2.7 V) and (**b**) aged at 85 ${}^{\circ }$C and 85% humidity conditions on the LED chips (20 mA, 2.7 V) [70].

The solution of inorganic NCs embedded in inorganic shells proves the best reliability. Li et al. proposed a strategy to synthesize ceramic-like stable and highly luminous CsPbBr_3_ NC through template-limited solid-state synthesis and in situ encapsulation based on the strategic disintegration of silicon molecular sieve (MS) templates at high synthesis temperatures [70]. The synthesis process is a solid state reaction at high temperature without organic solvents and organic ligands. The stability of perovskite NCs coated by molecular sieve was tested at high temperature (HT 85 ${}^{\circ }$C) and high humidity (HH 85%). It can be seen from Figure 27 that after 168-h aging, the luminous intensity of CsPbBr_3_−SiO_2_−HF basically remains unchanged, while the luminous intensity of ceramic Sr_2_Sio_4_:Eu_2_^+^ green phosphor decreases to 80% of the initial luminous intensity. Room remains for further improvement as the particle size should be reduced to sub-micron to incorporate with the micro-LED chip.

**Study on low dimensional PNC**. So far, the most stable one should be pure inorganic perovskite. According to the latest research progress of stability improvement strategy, the pure inorganic PNC coated by inorganic silica shows the best stability. According to the different connection modes of PbX_64_^−^ octahedron, lead-based perovskite can be divided into zero dimensional (0d), two-dimensional (2D) and three-dimensional (3D) structures. It has been described that 3D perovskite is prone to ion migration and quantum dot degradation due to defects, especially at the grain boundary. However, in the low dimensional NCs, there is almost no grain boundary, and the process of ion migration is different from that of 3D perovskite. According to our recent research [71], activated ion migration can promote the degradation of quantum dots, in which stable ion supply is needed to modify the perovskite / external contact interface. Therefore, the organic layer is inserted into MQW perovskite LED as the barrier of ion movement, which successfully inhibits ion migration and improves the stability. In addition, we observed that the capacitance technique is helpful to understand the migration of ions to the external electrode, because the capacitance in the low-frequency region is related to the ion migration and the double-layer polarization of the electrode. The measurement results of MQW perovskite shows that the starting frequency of ion migration is 0.1 Hz, which is three orders of magnitude lower than 3D CH_3_NH_3_PbI_3_−xClx perovskite (100 Hz). The study of electric absorption also shows that the activation energy of ions increases significantly without interface modulation. The in situ PL imaging of ions under applied bias shows that the movement of ions is obviously blocked. The activation energy Ea of the device is 0.48±0.09 ev by averaging the measurements of four different devices. Previous studies on CH_3_NH_3_PbI_3_−xClx and FAPbI_3_ perovskite have found that the activation energy of iodine ion/vacancy is mostly in the range of 0.1–0.3 ev. Therefore, the increase of activation energy means that the ion migration in MQW perovskite films has a larger potential barrier. This enhanced barrier is due to the inhibition of ion migration by the insertion of hydrophobic organic cations. Considering the vertical orientation of MQW perovskite relative to the substrate and the use of vertical device geometry, MQW structure also seems to reduce the level of ion migration parallel to MQW. This shows that the long organic molecules in the quasi two-dimensional materials also passivate the grain boundaries in the polycrystalline films and hinder the migration of ions or vacancies.Combining the basic properties of colloidal LHP-NCs and block 2D perovskite to realize multidimensional 3D/2D LHP-NCs has been proved to be an interesting research direction [72].

## 8. Applications of MHP-NCs

The current drawback in stability of MHP-NCs cannot diminish the research interests on its incorporation with displays. It is reasonable that current studies are mostly focused on the methodologies for improving the stability. A robust packaging is crucial to prevent the encroaching from ambient [6,73,74,75].

The excellent polarization characteristics of MHP significantly improve the backlight efficiency, indicating a bright application prospect in LCD. Wu’s team summarized two new trends in QD-LCD: (1) replacing traditional color filters with QD arrays; (2) emerging quantum rod (QR) enhanced backlighting [76]. As an important part of LCD, the design of the backlight affects the color gamut, optical efficiency, viewing angle, etc. With the continuous development of color gamut evaluation indicators, the development of new narrow-band transmitters has become increasingly critical. However, there is still a lack of a stable and narrow-band red down conversion layer, therefore, a hybrid backlight system is preferred [77]. In addition to careful thermal management, a tradeoff between life and brightness is also needed when High Dynamic Range(HDR) is required [78].

**Figure 28:**
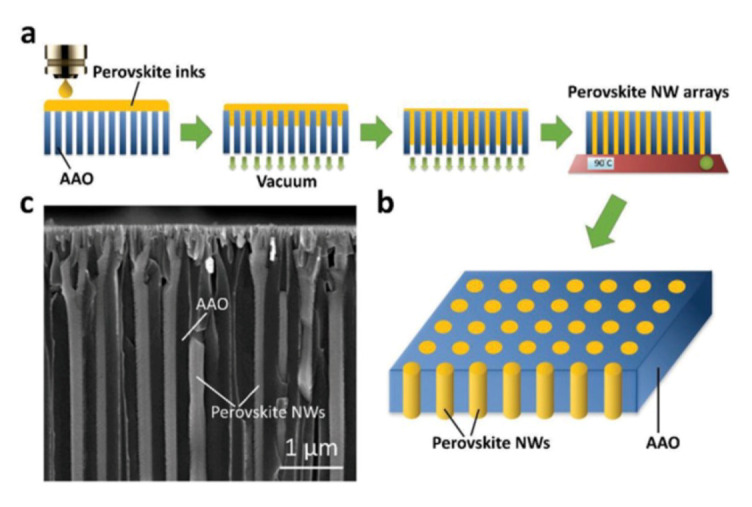
Fabrication process and characteristics of MHP-NWs. (**a**) MHP-NWs fabrication based on inkjet printing. (**b**) Schematics of MHP-NWs encapsulated in AAO nanopores. (**c**) The SEM image of MHP-NWs. Reproduced from [73].

**Figure 29:**
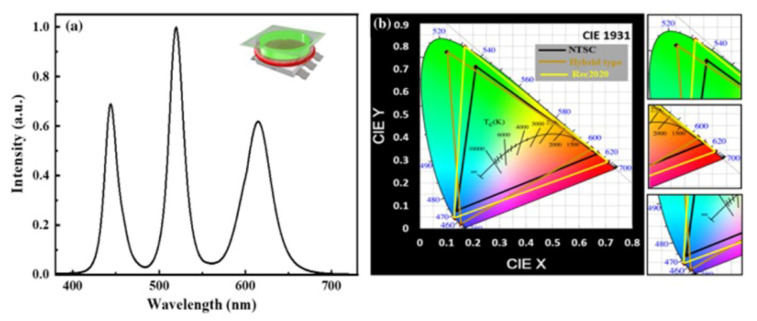
(**a**) the EL spectrum and (**b**) color gamut of hybrid-type white-LEDs. The inset in (**a**) shows the device structure. CsPbBr_3_ and CsPbBr_1.2_I_1.8_ function as down-conversion layers for green and red, respectively, which are excited by blue light. Reproduced from [74].

Lin et al. proposed a novel packaging structure to obtain high polarization effect. In this technique, MHP-NWs are encapsulated in anodic aluminum oxide (AAO) nanopores through inkjet printing technology [73]. The fabrication process is shown in Figure 28, under the controlled vacuum, MHP-NWs of well ordered and high crystalline quality are manufactured successfully. The emission wavelength and FWHM are 540 nm and 22 nm, respectively. Owing to the well aligned AAO nanopores structure, the MHP-NWs have better stability and higher degree of linear polarization of 0.84 compared to the traditional flat Al_2_O_3_ structure. Meanwhile, the MHP-NW arrays exhibit high EQE of 21.6%. To achieve display with high color quality, Prof. Kuo et al. innovatively demonstrated a hybrid-type MHP-NC structure to fabricate a white LED with high stability and high color gamut, which can overcome the shortcomings caused by only pure solid or liquid MHP-NC structure [74]. As shown in Figure 29a, the hybrid-type MHP-NCs are successfully excited by the blue LED, and the device structure is shown in the illustration, MHP-NCs are sealed in a glass, which effectively protect the MHP-NC from heating. Meanwhile, the hybrid-type white LED exhibits excellent color gamut with 122% of NTSC and 91% of Rec. 2020 standards, as shown in Figure 29b.

**Figure 30:**
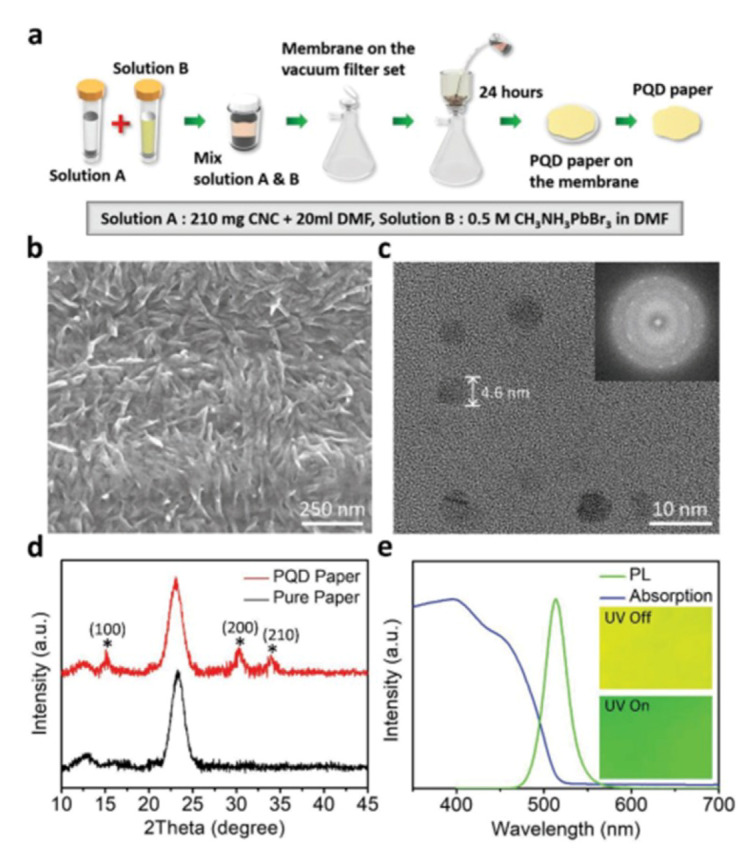
Process flow and morphology of the PQD paper. (**a**) fabrication flow of the PQD paper. (**b**) SEM image of the obtained PQD paper. (**c**) TEM image of the PQD paper. (**d**) comparation between PQD paper and pure CNCs paper XRD pattern. (**e**) Absorption and emission spectra of PQD paper [6].

It is crucial to simultaneously maintain high luminous efficiency and high stability for advanced lighting application. A lot of attempts were made in recent studies. He et al. uses MHP-polymer composite powder and film to emit different colors as down-converters, which can optimize visual energy efficiency, color rendering quality and circadian rhythm effect of the light source at the same time, preparing high-performance adjustable solid-state lighting source [79]. Kang et al. achieve a high luminous efficiency (124 lm/W) and wide color gamut (123% of NTSC) white LED via innovative MHP-NC paper technique [6]. Through filtering the mixed cellulose NCs and MHP solution under vacuum condition, MHP-NC paper is manufactured eventually, as shown in Figure 30a. The morphology and structure of the obtained MHP-NC paper is vividly presented in Figure 30b–d. Owing to the cellulose MHP-NC, the MHP-NC paper exhibits obvious entanglement phenomenon and the MHP-NC is well crystallized and capped at the same time, which could not only enhance the PL intensity and stability but also extend the lifetime (240 h within only 12.4% droop). The MHP-NC paper can be well excited by the blue LED and the emission wavelength and FWHM are 518 nm and 28 nm, respectively, as shown in Figure 30e.

**Figure 31:**
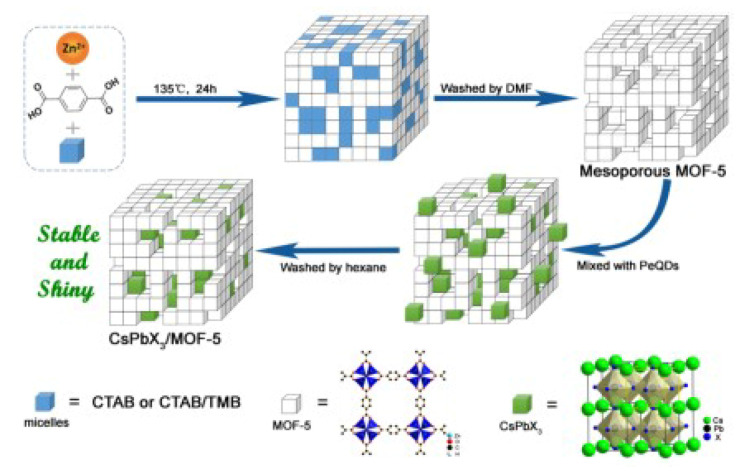
Fabrication of CsPbX_3_/MOF-5 composites. Reproduced from ref. [75].

Ren et al. proposed a zinc-based metal organic framework (MOF-5) structure [75]. The schematic manufacture process is shown in Figure 31, the all-inorganic CsPbX_3_ NCs are deposited into the mesoporous MOF-5 crystals. This packaging method can effectively alleviate the influence of light and heat. The fabricated white LED has high color stability even though under different driving current (10 mA–200 mA). Meanwhile, it has a wide color gamut of 124% NTSC, which makes it has a bright future in the high quantity lighting application.

Instead of sealing the MHP-NCs in some substrate materials, another solution to enhance protection is to absorb the MHP-NCs onto the surface of some host particle, which is stable in itself, and by this way the MHP-NCs can be protected by the nanocomposites. Li et al. first prepared heterogeneous nucleation-grown two-dimensional hexagonal boron nitride (h-BN) nanosheets at room temperature to stabilize CsPbBr_3_ PQDs [80].

**Figure 32:**
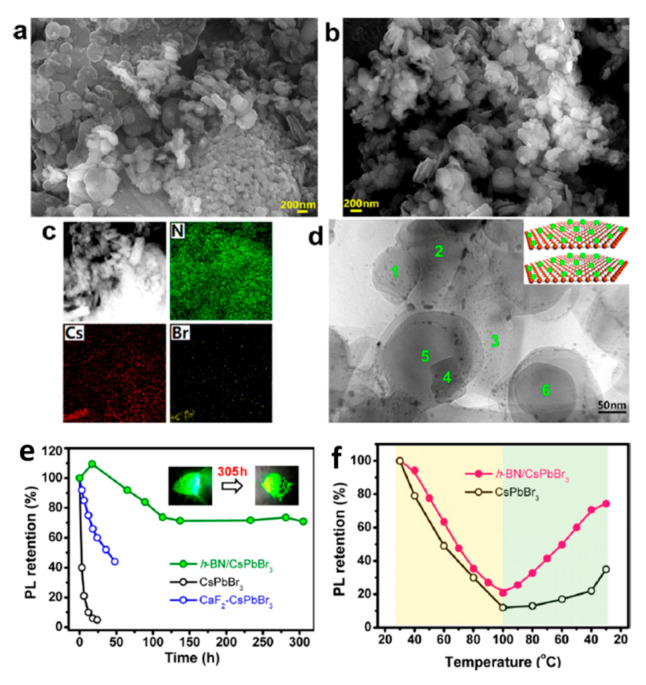
(**a**,**b**) SEM images of (**a**) pure h-BN and (**b**) h-BN/CsPbBr_3_ composite powder. (**c**) Areal distribution of N, Cs, and Br elements in h-BN/CsPbBr_3_ composite powder. (**d**) HRTEM image of h-BN/CsPbBr_3_ composite powder (inset illustrates the dispersion of CsPbBr_3_ PQDs on h-BN nanosheet surfaces). (**e**) Humidity stability and (**f**) thermal stability of h-BN/CsPbBr_3_ composite powder. Reproduced from [80].

As shown in Figure 32, equipped with high specific surface area and abundant mesopores of the h-BN nanosheets, cubic CsPbBr_3_ PQDs are attached to the surface of the h-BN nanosheets, forming h-BN/CsPbBr_3_ NC nanocomposites. Based on this structure, first, the nanosheets stacked in the h-BN/CsPbBr_3_ NC nanocomposites prevent water molecules from attacking the NC, and have a spatially restrictive effect on the growth and decomposition of the NC. Secondly, 2D h-BN nanosheets are excellent thermal conductors. It can effectively reduce the thermal stress inside h-BN/CsPbBr_3_ NC nanocomposites. Thirdly, the relatively strong van der Waals force between NCs and 2D nanosheets can prevent random movement of NC. Hence, due to this unique 2D structure and excellent thermal conductivity of h-BN nanosheets, h-BN/CsPbBr_3_ NC nanocomposites have demonstrated significant enhancement of humidity stability and thermal stability.

**Figure 33:**
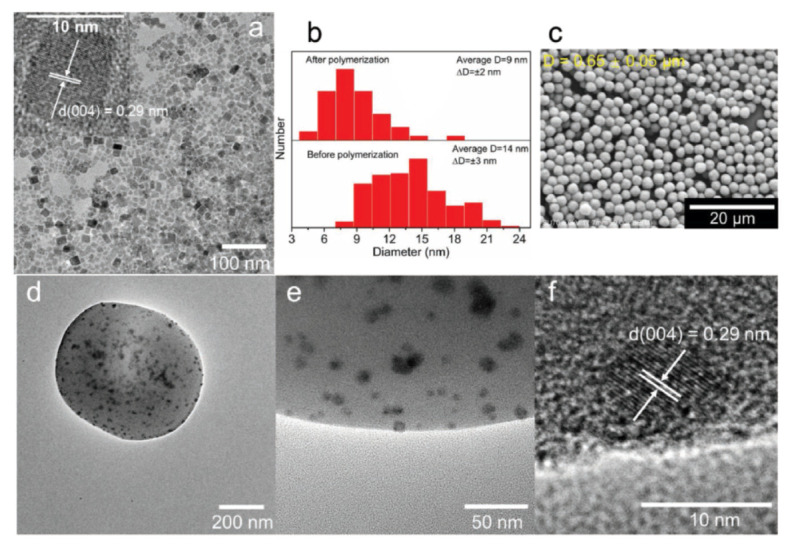
(**a**) TEM image of MAA-CsPbBr_3_ PQDs. (**b**) Histograms of lateral size distribution of MAA-CsPbBr_3_ PQDs before and after polymerization. (**c**) SEM image of MAA-CsPbBr_3_@PMMA composite microspheres. (**d**–**f**) TEM images of MAA-CsPbBr_3_@PMMA composite microspheres at different magnifications. Inset in (**a**) is the corresponding high-resolution TEM image of a selected MAA-CsPbBr_3_ PQD. Reproduced from [81].

Cai et al. prepare the composite microspheres of polymethyl methacrylate (PMMA) loaded with CsPbBr_3_ PQDs (CsPbBr_3_ @ PMMA) by in situ polymerization of methyl methacrylate in hexane in the presence of CsPbBr_3_ NC and stabilizer [81]. Due to the strong coordination interaction between the Pb ion on the surface of NC and the carbonyl group (C=O) of PMMA, NCs can be gradually incorporated as the microspheres grow, making them uniformly and firmly dispersed in the microspheres, shown in Figure 33. In addition, CsPbBr_3_ @ PMMA microspheres show adjustable size and narrow size distribution. Hence, with the protective effect of hydrophobic PMMA microspheres, the embedded CsPbBr_3_ NC reveals improved the water resistance and storage stability. This improvement makes CsPbBr_3_ @ PMMA as a promising material in the field of micro-LED display.

## 9. Conclusions

In conclusion, MHP-NCs, as a new type of semiconductor luminescent materials, have demonstrated the promising prospect in the field of lighting and display in the near future. Yet, MHP-NCs are surrounded by a large specific surface defect area, which results in non-radiative recombination. In addition, due to this large surface-to-volume ratio, external factors, viz. oxygen, water, ultraviolet light, and heating effect, especially the combination effect, give rise to the instability of MHP-NCs through different mechanisms. This degradation process has become the major challenges towards the large-scale commercial applications. To solve this stability problem, component regulation, surface passivation, coating with polymer/inorganic shells and utilizing low dimensional NCs, has proved to effectively reduce the defect state and prevent/reduce the interaction of MHP-NCs with water, oxygen, ultraviolet light and heating effect in the environment.

In terms of the future trend on the aspect of stability of MHP-NCs and their large application in lighting and display, seeking low-cost, mild synthesis conditions and good stability of the polymer matrix and MHP-NCs, deserves more attention from researchers. Yet, it is worth noting that each single method cannot prohibit all the degradation as we have presented previously. Therefore, besides the modification of the perovskite absorber layer itself, it is also critical to improve packaging technology for the reliable operation of the whole devices. The development history of silicon and organic optoelectronic devices has provided the valuable lessons on the encapsulation of devices against light soaking, oxygen and moisture. A proper industrial encapsulation strategy enables the large area MHP-NCs-based device to be undertaken under an ambient environment for more than several years. However, there is still a long way to seek and test the optimum sealing materials, encapsulation packaging layer, edge sealing technique, etc.

In addition, in order to promote the practical application of MHP-NCs-based devices, more bias tests under different ambient conditions are still required. Currently, industry standard damp heat and thermal cycling tests were applied for the stability test of perovskite photovoltaic devices [82,83]. While the characterization protocols, particularly the standard for operational stability for perovskite LED is still under debate [84]. Hence, it is urgent to make the standard for MHP-NCs-based lighting and display devices for the whole research community.
